# Crowdsourcing the Citation Screening Process for Systematic Reviews: Validation Study

**DOI:** 10.2196/12953

**Published:** 2019-04-29

**Authors:** Nassr Nama, Margaret Sampson, Nicholas Barrowman, Ryan Sandarage, Kusum Menon, Gail Macartney, Kimmo Murto, Jean-Philippe Vaccani, Sherri Katz, Roger Zemek, Ahmed Nasr, James Dayre McNally

**Affiliations:** 1 Faculty of Medicine University of Ottawa Ottawa, ON Canada; 2 Department of Pediatrics Children’s Hospital of Eastern Ontario Ottawa, ON Canada; 3 Faculty of Medicine University of British Columbia Vancouver, BC Canada; 4 Department of Pediatrics British Columbia Children's Hospital Vancouver, BC Canada; 5 Clinical Research Unit Children’s Hospital of Eastern Ontario Research Institute Ottawa, ON Canada; 6 Department of Anesthesiology and Pain Medicine Children’s Hospital of Eastern Ontario Ottawa, ON Canada; 7 Department of Otolaryngology Children’s Hospital of Eastern Ontario Ottawa, ON Canada; 8 Department of Emergency Medicine Faculty of Medicine Ottawa, ON Canada; 9 Division of Pediatric Surgery Children’s Hospital of Eastern Ontario Ottawa, ON Canada

**Keywords:** crowdsourcing, systematic reviews as topic, meta-analysis as topic, research design

## Abstract

**Background:**

Systematic reviews (SRs) are often cited as the highest level of evidence available as they involve the identification and synthesis of published studies on a topic. Unfortunately, it is increasingly challenging for small teams to complete SR procedures in a reasonable time period, given the exponential rise in the volume of primary literature. Crowdsourcing has been postulated as a potential solution.

**Objective:**

The feasibility objective of this study was to determine whether a crowd would be willing to perform and complete abstract and full text screening. The validation objective was to assess the quality of the crowd’s work, including retention of eligible citations (sensitivity) and work performed for the investigative team, defined as the percentage of citations excluded by the crowd.

**Methods:**

We performed a prospective study evaluating crowdsourcing essential components of an SR, including abstract screening, document retrieval, and full text assessment. Using CrowdScreenSR citation screening software, 2323 articles from 6 SRs were available to an online crowd. Citations excluded by less than or equal to 75% of the crowd were moved forward for full text assessment. For the validation component, performance of the crowd was compared with citation review through the accepted, gold standard, trained expert approach.

**Results:**

Of 312 potential crowd members, 117 (37.5%) commenced abstract screening and 71 (22.8%) completed the minimum requirement of 50 citation assessments. The majority of participants were undergraduate or medical students (192/312, 61.5%). The crowd screened 16,988 abstracts (median: 8 per citation; interquartile range [IQR] 7-8), and all citations achieved the minimum of 4 assessments after a median of 42 days (IQR 26-67). Crowd members retrieved 83.5% (774/927) of the articles that progressed to the full text phase. A total of 7604 full text assessments were completed (median: 7 per citation; IQR 3-11). Citations from all but 1 review achieved the minimum of 4 assessments after a median of 36 days (IQR 24-70), with 1 review remaining incomplete after 3 months. When complete crowd member agreement at both levels was required for exclusion, sensitivity was 100% (95% CI 97.9-100) and work performed was calculated at 68.3% (95% CI 66.4-70.1). Using the predefined alternative 75% exclusion threshold, sensitivity remained 100% and work performed increased to 72.9% (95% CI 71.0-74.6; *P*<.001). Finally, when a simple majority threshold was considered, sensitivity decreased marginally to 98.9% (95% CI 96.0-99.7; *P*=.25) and work performed increased substantially to 80.4% (95% CI 78.7-82.0; *P*<.001).

**Conclusions:**

Crowdsourcing of citation screening for SRs is feasible and has reasonable sensitivity and specificity. By expediting the screening process, crowdsourcing could permit the investigative team to focus on more complex SR tasks. Future directions should focus on developing a user-friendly online platform that allows research teams to crowdsource their reviews.

## Introduction

### Systematic Reviews and their Challenges

Systematic reviews (SRs) are often cited as the highest level of evidence available as they involve the identification and synthesis of all published studies on a topic [1]. Moreover, given the rise in the volume of primary literature, clinicians, scientists, and policy makers increasingly rely on SRs to inform decision making on important issues [2]. Maintenance of a continuous stream of up-to-date, high-quality evidence is important for optimal patient care and proper utilization of health care resources [3-7]. Unfortunately, it is more and more challenging for individuals and small teams to complete SR procedures in a reasonable time period [8-11]. To complete an SR, investigators need to manage thousands of potentially relevant citations, remove duplicates, screen abstracts for eligibility, download manuscripts, independently review full texts, resolve conflicts regarding eligibility, assess quality, extract and analyze data, and author a manuscript [8]. Consequently, there is significant interest in novel methodological approaches that improve the feasibility and completion of knowledge synthesis efforts and also avoid the scenario where investigators choose less than optimal search and screening strategies to maintain feasibility [12,13].

### Crowdsourcing in Science

Crowdsourcing has been postulated as a potential solution to address the barriers to efficient completion of SRs [14]. Crowdsourcing is “the practice of obtaining participants, services, ideas, or content by soliciting contributions from a large group of people, especially via the Internet” [15,16]. From tracking soil quality [17] and classifying galaxies [18] to identifying the three-dimensional (3D) configuration of complex protein structures [19], crowdsourcing has been studied and validated in other scientific areas. More recently, the medical field has seen increased application of crowdsourcing approaches to a wide range of problems ranging from funding research [20] to disease diagnosis (eg, Cell Slider [21]). In recent years, a small number of research groups have proposed and even evaluated crowdsourcing certain SR tasks [14,22-24]. These studies mainly focused on abstract screening, and to our knowledge, no previous research has studied the crowd’s capacity for full text retrieval and review.

### Objectives

The feasibility objective of this study was to determine whether it was possible to recruit an online crowd to perform and complete abstract and full text screening for SRs. The validation objective was to assess the quality of work performed by the crowd when compared with the gold standard expert approach, both in regards to the sensitivity for eligible citations and the potential work performed for the investigative team.

## Methods

### Study Design

This study was conducted at the Children’s Hospital of Eastern Ontario (CHEO), a teaching hospital affiliated with the University of Ottawa. Similar to previous studies in this field [22], and as per the CHEO Research Ethics Board, this study was not considered as research on humans, and as such, ethics approval was not required. The project description clearly stated that crowd members were not eligible for authorship and that their contribution was part of a research study validating crowdsourcing as a new methodology in the area of SRs. On sign-up and log-in, the crowd was provided with both privacy policy and terms of use documentation, designed in consultation with the CHEO privacy lawyer (Multimedia Appendix 1).

This study was a prospective quantitative study evaluating the feasibility and validity of crowdsourcing essential components of an SR, including abstract screening, document retrieval, and full text assessment. For the validation component, performance of the online volunteer crowd was compared with citation review through the accepted, gold standard, trained expert approach. Results are reported according to the Standards for Reporting of Diagnostic Accuracy Studies guidelines for diagnostic accuracy studies [25] (Multimedia Appendix 2).

### Study Outcomes

The primary outcome for the feasibility component was the number of citations that achieved the target number of independent assessments. Consistent with our initial pilot study, feasibility success was a priori defined as achieving a minimum of 4 independent assessments per citation [23]. The primary outcome for the validation component was the ability of the crowd to identify and retain eligible studies at the abstract level (sensitivity). For the validation component, secondary outcomes included the crowd’s overall sensitivity after full text review of retained abstracts and the work performed. Work performed was defined as the percentage of all citations that were excluded by the crowd and did not require assessment by the investigative team at abstract or full text levels. To allow comparison with other studies, specificity was also calculated. Individual reviewer’s performance represented an exploratory outcome.

### Sample Size and Power

For the purpose of the sample size calculation, the crowd retention of true positives (ie, the sensitivity) was assumed to be 95% at the abstract screening level. Under this assumption, the sample size was selected so that the lower end of the 95% CI for sensitivity would be no less than 90%. Using a Wilson score CI, this would be the case if 142 abstracts were retained by crowd members out of a total of 150 abstracts deemed eligible by expert screeners (95% sensitivity). Thus, a sample size of 150 abstracts was selected.

### Systematic Review Selection and Details

Potentially eligible SRs included those initiated during 2016 and not anticipated to be published before the end of the 2017 calendar year (to prevent crowd members from accessing the published data with lists of eligible papers). The reviews selected covered the areas of anesthesiology, cardiology, emergency medicine, endocrinology [13], respirology, and general surgery [26] (Table 1). We targeted a wide range of topics with the intention of making the results more generalizable and increasing the likelihood that a potential crowd participant would identify a topic of interest. For each SR, the principal investigator was asked to provide the following: (1) inclusion and exclusion criteria and (2) the final list of citations determined to be eligible by their expert reviewers (true positives). In some circumstances, the investigative team provided screening criteria that differed slightly from their original review. In this circumstance, study authors NN and DM reviewed the true positives against criteria presented to the crowd, and any study not meeting the eligibility criteria provided to the crowd was removed from the true positive list. For SRs exceeding 1000 citations, smaller subsets were chosen, ensuring a reasonable pool of true positives (Table 1).

### Crowd Recruitment and Compensation

To qualify for participation, the individual needed to be both a nonexpert and a member of a large distributed crowd. To be considered a nonexpert, individuals had to confirm that they had not participated in the development of the protocol for the SR and had not received training sessions by the investigators on how to screen citations. For this initial feasibility study, we targeted the large online crowd or population of individuals with some postsecondary or postgraduate training, including undergraduate, medical students, residents, nurses, and other allied health specialists. We targeted this population for 2 reasons: (1) given the paucity of work on crowdsourcing SRs, it seemed appropriate to begin by evaluating the performance measures in a cohort with or receiving applicable science or health training and (2) similarly skilled and motivated individuals would be available and accessible at dozens of cities in Canada and hundreds through the world. Individuals were recruited by sending emails to (1) the hospital volunteer department, (2) University of Ottawa Medical School, (3) student interest groups at the 17 Canadian medical schools, and (4) health-related undergraduate student groups in 22 universities across Canada. Promotional material was designed by CHEO Media House (Multimedia Appendix 3). As a resource for those who might want to recruit a crowd with similar characteristics and motives to perform a large SR, we have provided an example copy of the email sent (Multimedia Appendix 3). Compensation was limited to the potential for a gift card (Can $100) for the top 3 crowd members in each review (highest number of citations screened accurately). Furthermore, we offered crowd members the possibility to connect them with CHEO investigators performing an SR and seeking to grow their research team. For reviews that did not attain the minimum of 4 assessments per citation at either level after 2 months, additional gift cards were offered. In total, 26 gift cards were distributed among 22 crowd members.

### Crowdsourcing Website Development and Overview of the Platform Function

To complete this study, we used the CrowdScreenSR citation screening platform, as previously described [23]. The website was adopted by the CHEO Research Institute in 2016 and was concurrently used by 4 of the 6 investigative teams for completion of their SRs using the gold standard or expert approach. Crowd members had unique usernames and passwords, allowing separate tracking and evaluation of progress, work performed, and performance. Demographic data were collected on crowd members, including the level of training, research experience, participation in previous SRs, and number of publications. Crowd members were instructed to select only the highest level of training in progress or completed. Initially, each crowd member was given access to a demonstration module to help familiarize them with the website functioning. Initially, all 6 reviews were shown to the crowd, along with a description of the goal of the study and its eligibility criteria. For each SR, a training set of 10 citations, including 2 to 3 true positives, was used to familiarize the crowd with both the SR eligibility criteria and platform. During this training set, immediate feedback was provided on whether the crowd member’s assessment of the citation was accurate. Crowd members who completed the training set were given access to the full set of citations for that review (regardless of their performance). A minimum goal of at least 50 citations was set, with crowd members offered the flexibility of screening as many citations as desired. For both abstract and full text screening levels, the crowd members were instructed to place citations into 1 of the 3 groups: (1) retain, (2) exclude, or (3) no assessment (not comfortable assessing this citation). When a citation was categorized as exclude, the crowd member was further prompted to provide which eligibility criteria were not met. We have aimed to achieve at least four assessments per citation at each of abstract and full text levels, with no predefined maximum. Abstract-level screening started on January 7, 2017, and was completed on April 23, 2017. Retrieval of manuscripts, PDF upload, and full text screening continued until September 3, 2017. Start dates for each of these phases were chosen at the beginning of university trimesters to maximize crowd members participation.

**Table 1:** Description of systematic reviews.

Systematic review^a^	Description	Total citations^b^, N	Validation study^c^, N	Eligible citations^d^, N (%)
Anesthesiology^e^	A systematic review of preoperative screening for factors associated with postoperative critical respiratory events in children undergoing elective adenotonsillectomy	5458	300	29 (9.7)
Cardiology^f^	A scoping review of all randomized controlled trials in pediatric cardiology	7540	490	71 (14.5)
Emergency	A systematic review of studies on concussion education and outcomes for children	513	503	9 (1.8)
Endocrinology^g^	2017 update of a previously published systematic review on high-dose supplementation of vitamin D in children [13,23]	201	201	30 (14.9)
Respirology	A systematic review of studies on predictors of positive airway pressure adherence at home among children with sleep-disordered breathing	277	265	23 (8.7)
Surgery	A systematic review of studies on asymptomatic antenatal diagnoses of congenital pulmonary airways malformation that describe natural history of the disease and future symptoms [26]	574	564	16 (2.8)

^a^Total of 6 systematic reviews and 2323 citations were included. 178 (7.7%) of citations were identified as eligible by the experts (ie, true positives).

^b^Total number of citations identified by the search strategy.

^c^Number of citations included in the validation study, after excluding the 10 citations used as a training set.

^d^Eligible citations as identified by the experts (ie, true positives).

^e^A random sample of 300 citations was selected and enriched with up to 30 eligible citations.

^f^A random sample of 500 citations was selected.

^g^Given the limited number of citations, the 10 training set citations were selected from the original publication.

### Advancement to Full Text Screening

**Figure 1:**
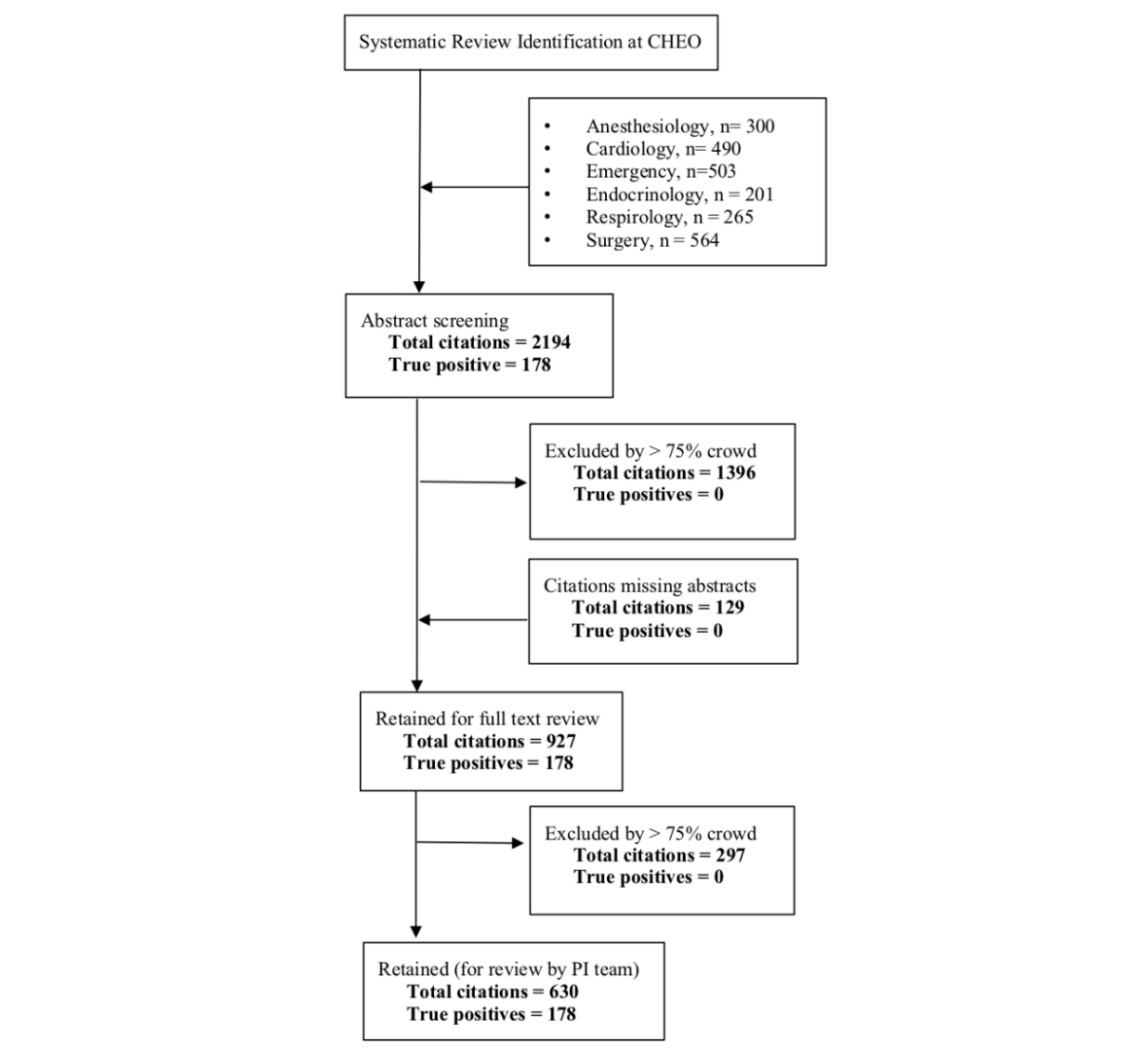
Study flow diagram. To focus the study on the crowd’s capacity to assess abstracts and not title screening, citations with missing abstracts (129) were removed. These citations were later added to the full text screening stage, along with any citation that did not receive higher than our a priori exclusion threshold of 75% at the abstract screening level. True positives reflect the number of citations that were identified as eligible by the experts. CHEO: Children’s Hospital of Eastern Ontario; PI: principal investigator.

To focus on the crowd’s capacity to assess abstracts, citations with missing abstracts were automatically pushed forward to full text retrieval and review. In addition to those with missing abstracts, citations where greater than or equal to 25% of the crowd assessed as eligible were retained for use in the assessment of crowd performance at full text review (Figure 1).

### Validation of Crowd Performance

For the purpose of the analysis, different thresholds for citations’ exclusion were tested. Specific exclusion cut-offs (75% and 100%) were prioritized in the analysis as these were both tested and performed well in our previous study [23]. To allow comparison with another recently published study [22], we also considered the 50% exclusion threshold (Multimedia Appendix 4). Finally, a range of exclusion thresholds between 0% and 100% were tested and presented graphically. Using the 0% cut-off was the least conservative approach, where a citation was excluded if any crowd member opted to exclude. On the other end, the 100% cut-off was the most conservative, and a citation was only excluded if every crowd member chose to exclude. Measures of individual crowd members’ performance were completed as an exploratory analysis and were limited to those crowd members having completed a minimum of 50 citation assessments. This cut-off was established a priori, as crowd members were asked to complete a minimum of 50 citations to increase the chance that the subset of citations assessed would contain at least a few eligible papers.

### Data Analysis

Data analysis was performed using SAS (version 9.4; SAS Institute, Cary, NC, USA). Figures were generated using GraphPad Prism (version 8.0; GraphPad Software, Inc, La Jolla, CA, USA). Fisher exact and Pearson Chi-square tests were used to compare characteristics of crowd members who proceeded to complete the minimum 50 citations with those who did not. Wilson score method was used to calculate 95% CIs for sensitivity, specificity, and work performed. The McNemar 1-tailed test was used to compare sensitivity and work performed between different exclusion thresholds. As a more stringent threshold for excluding a paper can only increase the sensitivity, a 1-tailed test was used.

## Results

### Crowd Description

A total of 313 individuals signed up on the CrowdScreenSR website. None of those were deemed ineligible based on our criteria. Of the 312 potential crowd members, 171 (54.8%) initiated at least one SR training set and 117 (37.5%) completed the training set and commenced abstract screening. Of these 117 crowd members, 71 (60.7%) completed 50 or more independent citations (Table 2). With regards to the crowd’s demographics, the most commonly selected answers were the highest level of training as undergraduate studies (131/312, 42.0%) and some prior research experience (220/312, 70.5%). One-third of the participants reported having been an author on a least one research publication (103/312, 33.0%), with only 1 in 5 citing previous involvement with SR research (65/312, 20.8%). Comparing those who proceeded to complete the minimum 50 citations with those who did not showed no statistically significant differences with respect to the level of training, prior research experience, publications of any kind, or involvement in SRs.

**Table 2:** Comparison of crowd members who proceeded to complete the minimum 50 citations with those who did not.

Crowd members		<50 assessments^a^, N (%)	≥50 assessments, N (%)	*P* value^b^	Total
Total reviewers		241 (77.2)	71 (22.8)	—^c^	312
**Background^d^**		—	—	.15	—
	Undergraduate studies	107 (44.4)	24 (33.8)	—	131
	Medical student	41 (17.0)	20 (28.2)	—	61
	Graduate studies	36 (14.9)	9 (12.7)	—	45
	Allied health professional	20 (8.3)	3 (4.2)	—	23
	Physician	7 (2.9)	3 (4.2)	—	10
	Other	4 (1.7)	3 (4.2)	—	7
**Research involvement^e^**		—	—	.08	—
	None	65 (27.0)	27 (38.0)	—	92
	Student	130 (53.9)	35 (49.3)	—	165
	Volunteer	81 (33.6)	23 (32.4)	—	104
	Coordinator	66 (27.4)	11 (15.5)	—	77
	Investigator	25 (10.4)	3 (4.2)	—	28
**Publications**		—	—	.23	—
	None	156 (64.7)	53 (74.6)	—	209
	1-3	57 (23.7)	14 (19.7)	—	71
	>3	28 (11.6)	4 (5.6)	—	32
**Systematic reviews experience**		—	—	—	—
	Involvement in a review	52 (21.6)	13 (18.3)	.62	65
	Leading a review	12 (5.0)	5 (7.0)	.55	17
	Publishing a review	38 (15.8)	12 (16.9 )	.85	50

^a^Minimum of 50 citations in a systematic review was requested from crowd members at the beginning of the study. Crowd members with 50 citations or more performed 98.8% (16,789/16,988) and 93.0% (7071/7604) of the abstract and full text assessments, respectively.

^b^Comparison between those who did less than 50 assessments and those who did 50 or more (Fisher test).

^c^Not applicable.

^d^Only 277 crowd members provided their background.

^e^Multiple choices can be selected by reviewers.

### Systematic Review Tasks Performed by Crowd (Feasibility)

#### Abstract Screening

**Figure 2:**
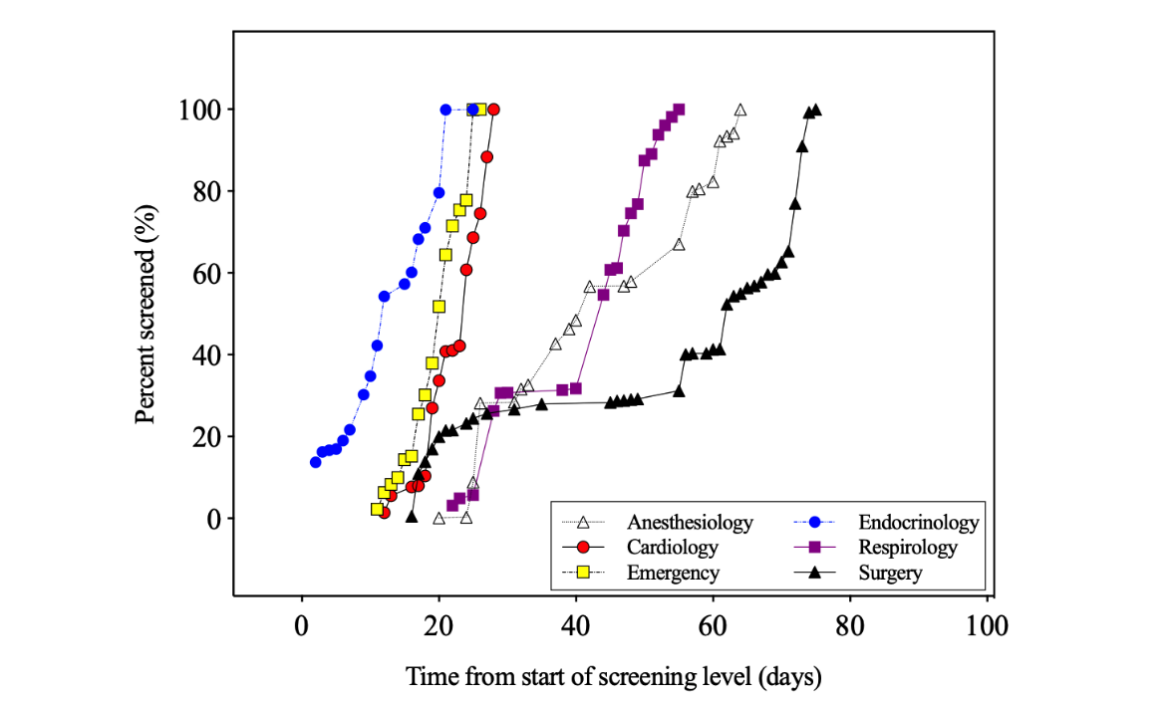
Time to review completion during abstract screening. Time required to complete the desired 4 assessments per citation at the abstract screening level. On day 61, additional incentives were offered for the surgery review.

Crowd members performed 16,988 abstract assessments on 2194 unique citations, and all of the citations met or exceeded the feasibility target of 4 independent assessments, with a median of 8 assessments per paper (interquartile range [IQR] 7-8; Multimedia Appendix 5). The time required to acquire 4 independent assessments per citation at the abstract level varied by review, with a median of 42 days (IQR 26-67; Figure 2). A total of 3 reviews were completed in less than a month: endocrinology (25 days), emergency (26 days), and cardiology (28 days). Of the remaining reviews, 2 (respirology and anesthesiology) required 55 and 64 days for completion, respectively. A total of 2 months after the project was launched, the 1 remaining review remained below the target of 4 assessments per paper (<50%). When the incentive was revised to a Can $100 gift card for any crowd member that completed all citations in that review, the project was completed in the subsequent 14 days.

#### Retrieval of Full Text

Following abstract screening, 927 papers were pushed to the full text level (Figure 1). Crowd members were able to successfully retrieve 83.5% (774/927) of the articles that progressed to the full text review phase. Of the 153 articles that were not retrieved by the crowd, 95% (145) were not open access and not available through the University of Ottawa.

#### Full Text Screening

**Figure 3:**
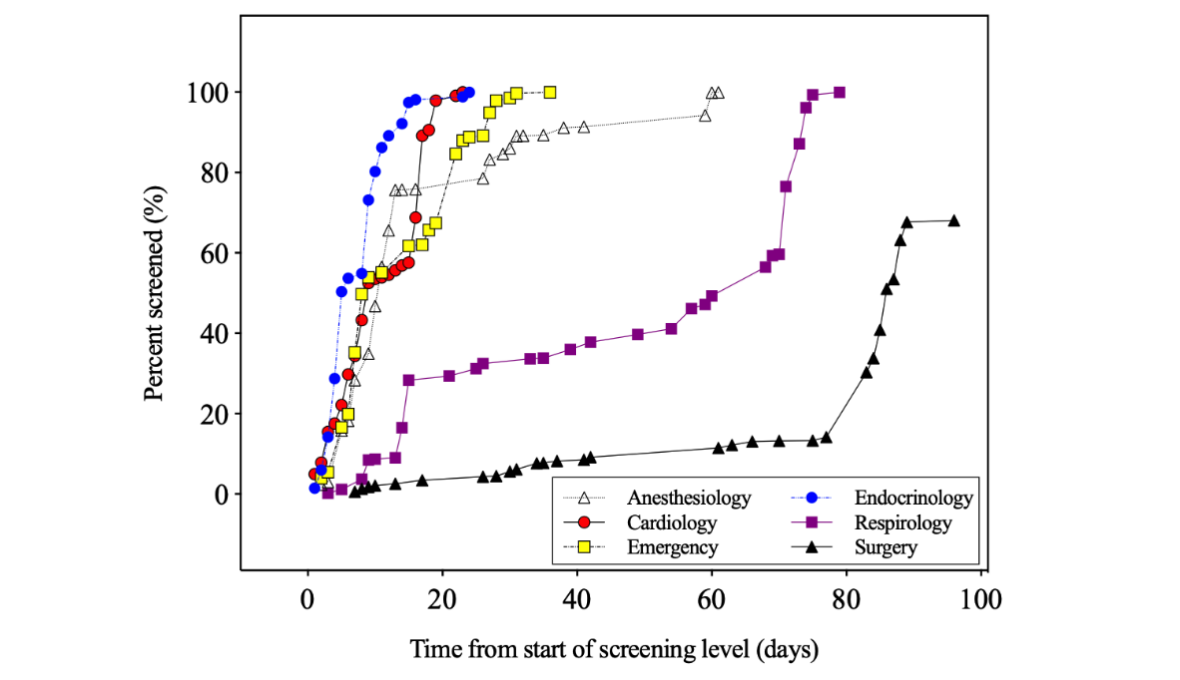
Time to review completion during full text screening. Time required to complete the desired 4 assessments per citation at the full screening level. Between days 58 and 77, reviewers were notified that the screening deadline is for day 90, and further incentives were offered for the anesthesiology, surgery and respirology reviews.

At full text review, the crowd members performed 7604 assessments on 927 unique articles (Multimedia Appendix 5). Of the 6 SRs, 5 achieved 4 assessments for all of their citations. Overall, median assessments number per citation was 7 (IQR 3-11). Full text review required a median of 36 days (IQR 24-70), with 1 review that remained incomplete after 3 months (Figure 3). In the first month, the crowd completed both the cardiology (23 days) and endocrinology (24 days), with emergency completed shortly thereafter (36 days). Anesthesiology was at 89.3% at the time and remained without significant progression until an email notifying the crowd that the remaining reviews were closing was sent on day 60, and the review was completed a day later. Additional $100 gift cards were offered for the other 2 SRs. With these efforts, the respirology review was completed after 79 days. The crowd did not complete full text review for the surgery SR, with only 1.4% (4/283) of citations above the 4-assessments threshold; 82 citations of those had only 2 assessments, and the other 197 citations had 3 assessments at the full text stage.

### Validation of the Crowd Performance—Abstract Level

**Figure 4:**
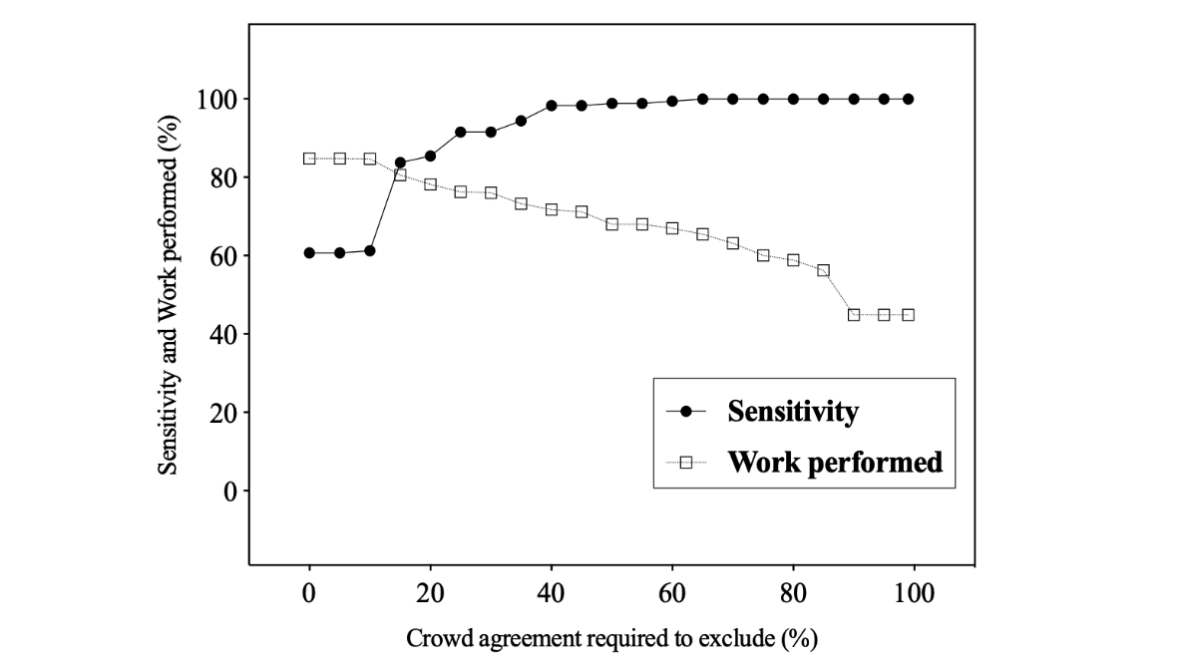
Sensitivity and work performed as a function of the exclusion threshold at the abstract level. A citation is excluded when the percentage of exclusion assessment is above the exclusion cut-off at the abstract level. Sensitivity is the percentage of eligible citations identified by the experts that were retained by the crowd. Work performed is the percentage of citations that were excluded by the crowd and did not require assessment by the investigative team at the abstract level.

When complete crowd member agreement at the abstract level was required for exclusion, sensitivity was 100% (95% CI 97.9-100) and work performed was calculated at 44.9% (95% CI 42.8-46.9; Table 3). Using the predefined 75% exclusion threshold, with citations excluded if more than 75% of the crowd agreed at the abstract level, sensitivity remained 100% and the work performed increased to 60.1% (95% CI: 58.1-62.1; *P*<.001). Finally, when a simple majority was required to exclude a citation, sensitivity decreased marginally to 98.9% (95% CI 96.0-99.7; *P*=.25) and the work performed increased to 68.0% (95% CI 66.1-69.9; *P*<.001). Sensitivity and work performed data were calculated for each of the individual SRs (Multimedia Appendix 6). Crowd specificity for abstract screening at 100%, 75%, and 50% exclusion thresholds was calculated as 48.6%, 65.1%, and 73.6%, respectively. Finally, the relationship between sensitivity and work performed after abstract screening at exclusion thresholds ranging from 0% to 100% is presented in Figure 4.

### Validation of the Crowd Performance—Full Text Level

**Figure 5:**
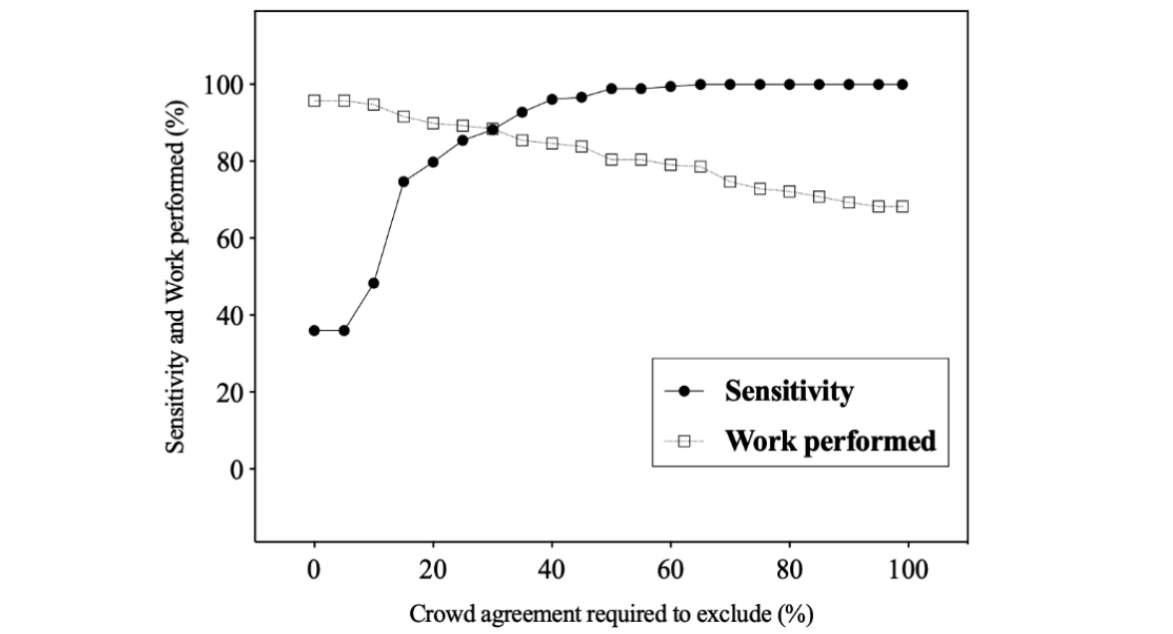
Sensitivity and work performed as a function of the exclusion threshold after abstract and full text screening. A citation is excluded when the percentage of exclusion assessment is above the exclusion cut-off at either abstract or full text screening. Sensitivity is the percentage of eligible citations identified by the experts that were retained by the crowd. Work performed is the percentage of citations that were excluded by the crowd and did not require assessment by the investigative team at abstract or full text levels.

Crowd’s performance was assessed after full text screening of retained abstracts. All eligible citations that were retained at the abstract level were also retained by the crowd at the full text level, and sensitivity remained the same based on the 3 exclusion thresholds (Table 3). When complete crowd member agreement at both levels was required for exclusion, work performed was calculated at 68.3% (95% CI 66.4-70.1). Using the predefined 75% exclusion threshold, with citations excluded if more than 75% of the crowd agreed at both the abstract and full text level, the work performed increased to 72.9% (95% CI 71.0-74.6; *P*<.001; Table 3). Finally, when a simple majority was required to exclude a citation, the work performed increased substantially to 80.4% (95% CI 78.7-82.0; *P*<.001). Sensitivity and work performed after screening both levels were calculated for each of the individual SRs (Multimedia Appendix 7). Crowd specificity after screening both levels at the 100%, 75%, and 50% exclusion thresholds were calculated as 73.9%, 78.9%, and 87.0%, respectively. Finally, the relationship between sensitivity and work performed at exclusion thresholds ranging from 0% to 100% is presented in Figure 5.

**Table 3:** Crowd’s sensitivity and work performed at different exclusion thresholds.

Crowd agreement required to exclude^a^		Sensitivity^b^		Work performed^c^		Specificity^d^	
		Mean (95% CI)	*P* value^e^	Mean (95% CI)	*P* value^e^	Mean (95% CI)	*P* value^e^
**Abstract level^f^**							
	=100%	100 (97.9-100)	.50	44.9 (42.8-46.9)	<.001	48.6 (46.5-50.7)	<.001
	>75%	100 (97.9-100)	(Ref^g^)	60.1 (58.1-62.1)	(Ref)	65.1 (63.0-67.1)	(Ref)
	>50%	98.9 (96.0-99.7)	.25	68.0 (66.1-69.9)	<.001	73.6 (71.7-75.4)	<.001
**Full text level^h^**							
	=100%	100 (97.9-100)	.50	68.3 (66.4-70.1)	<.001	73.9 (72.0-75.8)	<.001
	>75%	100 (97.9-100)	(Ref)	72.9 (71.0-74.6)	(Ref)	78.9 (77.2-80.6)	(Ref)
	>50%	98.9 (96.0-99.7)	.25	80.4 (78.7-82.0)	<.001	87.0 (85.5-88.4)	<.001

^a^Citations were excluded based on different thresholds.

^b^Sensitivity is the percentage of eligible citations, identified by the experts, that were retained by the crowd.

^c^Work performed is the percentage of citations that were excluded by the crowd and did not require assessment by the investigative team at the abstract level.

^d^Specificity is the percentage of ineligible citations, as identified by the experts, that were excluded by the crowd.

^e^*P* value compares sensitivity, work performed, or specificity to the respective value at the 75% threshold (McNemar test).

^f^Outcomes were measured after abstract screening. A citation was excluded if the percentage of assessments that excluded the paper at the abstract level was higher than the specified threshold.

^g^Ref: reference category.

^h^Outcomes were measured at the end of both screening levels. A citation was excluded if the percentage of assessments that excluded the paper at either abstract or full text levels was higher than the specified threshold.

**Table 4:** Individual crowd members’ performance.

Performance^a,b^	Abstract level (N=40)		Full text level (N=41)	
	Median (IQR^c^)	Range	Median (IQR)	Range
Assessments	306.5 (108.5-513.5)	16-2194	141 (72-206)	5-786
Sensitivity^d^	96.6 (92.0-100.0)	55.0-100.0	96.7 (89.6-99.0)	32.3-100.0
Specificity^e^	76.4 (66.2-92.8)	42.4-96.3	64.3 (58.5-73.8)	22.9-100.0

^a^Only crowd members who have completed 50 assessments or more in 1 review were included in this table. Crowd members with 50 citations or more performed 98.8% (16,789/16,988) and 93.0% (7071/7604) of the abstract and full text assessments, respectively.

^b^Results are provided per crowd member.

^c^IQR: interquartile range.

^d^Sensitivity is the percentage of eligible citations, identified by the experts, that were retained by the crowd member. It is based on 38 crowd members at the abstract level and 38 at the full text level. The remaining crowd members did not assess any eligible citations.

^e^Specificity is the percentage of ineligible citations, as discarded by the experts, that were also excluded by the crowd member.

### Individual Crowd Member’s Performance

In addition to crowd performance, we evaluated individual crowd member performance as an exploratory outcome in those users having completed a minimum of 50 citation assessments (Table 4). At the abstract level, these crowd members completed a median of 306.5 assessments (IQR 108.5-513.5) and performed 98.8% (16,789/16,988) of the assessments. Individual crowd member sensitivity was calculated as a median of 96.6% (IQR 92.0-100.0), with median specificity determined to be 76.4% (IQR 66.2-92.8). At the full text level, these crowd members completed a median of 141 assessments (IQR 72-206) and performed 93.0% (7071/7604) of the assessments. Individual crowd member sensitivity was calculated as a median of 96.7% (IQR 89.6-99.0), with median specificity determined to be 64.3% (IQR 58.5-3.8). Including crowd members who have completed less than the required minimum did not have any substantial differences on the results (Multimedia Appendix 8). Individual crowd member’s performance separated by SR is presented separately (Multimedia Appendix 9).

## Discussion

### Summary of Results

This study focused on crowdsourcing the citation review process and provides evidence suggesting both the feasibility of and the validity of this approach. First, using citations from 6 different SRs, we were able to establish that an online crowd was willing to assist with abstract screening, full text retrieval, and full text review. Importantly, this work also demonstrated that the online crowd showed a preference for certain reviews, with some reviews requiring incentives to attract crowd members and facilitate completion of abstract and full text screening. Second, through a comparison with the assessments performed by expert reviewers, we were able to demonstrate that the crowd had excellent sensitivity and performed more than 70% of abstract and full-text screening, depending on the threshold used for exclusion.

### Feasibility of Crowd Screening Systematic Reviews

Multiple health and science initiatives have recently proven that online individuals are willing and motivated to participate in crowdsourcing projects. In addition to Wikipedia, a well-known crowdsourcing initiative, FoldIt, is an excellent example, where over 57,000 individuals have participated in an online game working to predict protein 3D structures [19], outperforming both computational and experimental methods [27]. Other examples from the medical field have shown the crowd to be able to assess images of optic disks and diagnose diabetic retinopathy [28-30]. Similarly, in this study, we were able to recruit an online crowd of volunteers that was sufficiently sized to surpass the target for abstract screening, locate 83% of full text articles, and complete the full text assessment for 5 of the 6 reviews. This finding, when combined with the observations that the time to task completion was significantly different between reviews, with certain reviews requiring gift card incentives, suggests that feasibility may be specific to the crowd—review dyad. A crowd’s capacity to retrieve full text articles and screen them has not been evaluated previously, with related reports focusing solely on abstract screening. Although comparable published literature is limited, our results are consistent with other studies reporting that people are willing to perform SR tasks as either volunteers [23,24] or in exchange for payment [14,22]. For example, in a study by Mortensen et al [22], individuals working on the Amazon mTurk platform were paid to screen more than 1000 abstracts against the eligibility criteria for 4 different SRs of similar sizes, with the reviews completed in 5 to 17 days. The Cochrane Crowd initiative offers another great example supporting the feasibility of crowdsourcing [24,31]. Although published data are minimal on the Cochrane initiative, they have successfully organized an online community with thousands of individuals who have voluntarily screened over a million abstracts to identify those representing randomized controlled trials (RCTs) on humans. More recently, they have also evaluated having the crowd assist with individual Cochrane reviews, with online reports and abstracts demonstrating rapid completion of abstract screening (<5 days) [32]. Although review completion took slightly longer in our study because of the need to recruit a crowd de novo, the work from Amazon mTurk [14,22] and Cochrane [24] suggest even greater feasibility (ie, shorter times to review completion), given the immediate access to a large and sufficiently motivated crowd.

Self-reported information on training and research collected at participant sign-up in our study demonstrated that the majority were undergraduates or medical students with limited research experience. Although this approach does not allow us to comment on the performance of a more general online population, it is consistent with what has been observed and accepted by other successful crowdsourcing efforts in medicine and science. For example, surveys of crowdsourcing platforms such as Amazon mTurk and CrowdFlower have shown that crowd members are well educated, with around two-thirds having a college or advanced degree and a third being current students [33]. Furthermore, preliminary results from the Cochrane crowd suggest that more than 50% of crowd members worked in health-related fields [34]. On the basis of our results and those by Mortensen, it would now be reasonable to consider a study focused on the performance of the much large group of online workers without scientific or health training.

### Crowd Performance—Sensitivity

Although the ability to recruit an online crowd willing to perform SR tasks was the essential first step, it is of equal importance to understand crowd performance. Similar to studies evaluating other alternative methodologies with the potential to facilitate citation screening, we selected sensitivity as the most important performance outcome [35]. Although no consensus study (eg, delphi, survey) has defined the minimum acceptable sensitivity, 95% has become the industry standard in the field of automated text recognition research based on original studies by Cohen [11,36]. In our work, both of the a priori algorithms not only achieved sensitivities above 95% but the sample size also allowed us to exclude 95% from the 95% lower CI. When further reducing the crowd threshold to a majority (≥50% exclusions) to allow for comparison with the Mortensen study, the sensitivity fell only marginally to 98.9% because of the crowd exclusion of 2 studies. Inspection of these 2 publications identified that each abstract presented information on 2 different studies packaged into 1 manuscript, with the first study described not meeting eligibility criteria [37,38]. Our study is the first to assess the crowd’s ability to screen citations at the full text level. Similar to what is done currently by investigative teams, all articles that were retained at the abstract level at each of the 3 exclusion thresholds were moved to full text screening. The crowd showed high sensitivity at this level and did not miss any further eligible citations, even when only a simple majority was sufficient to exclude. In Mortensen’s study [22], the crowd’s sensitivity at the abstract level was compared against the gold standard approach of expert reviewers. Eligibility criteria were modified and slightly broadened for simplification purposes. The crowd sensitivity was lower than that of our study and varied between 86 to 93% using a threshold comparable with our 75%, and 71% to 89% when a simple majority was allowed to exclude an article.

### Crowd Performance—Work Performed

Although establishing high sensitivity is essential, crowdsourcing is only valuable if it effectively decreases the work required of the investigative team. In this study, our 2 a priori defined algorithms (100% and 75% exclusion thresholds) reduced the work required by the investigative team by approximately 45% to 60% after the abstract level. Allowing the crowd to screen the full text for citations retained at the abstract level significantly increased the work performed on behalf of the investigative team (70%). This additional 10% to 25% increase in work performed would translate to between 200 and 500 fewer full text articles to screen in an SR of 2000 citations. The crowdsourcing validation study by Mortensen presented gain (specificity) as a measure of work performed [22]. Their algorithm requiring 100% agreement to exclude a citation achieved gains between 68% and 87% across the 4 SRs and saved 90% of the cost of the gold standard experts’ approach. Using this definition, our gain was comparable and measured between 50% and 75% at the abstract level and further increased significantly to 75% to 85% after full text and depending on the exclusion threshold. It is important to note that the crowd has achieved high efficiency in both of these studies, despite slightly broadening the eligibility criteria.

### Performance of Individual Crowd Members

As an exploratory objective, this study also sought to understand the performance of individual uncurated volunteer crowd members, with the results suggesting the average participant user to have excellent sensitivity (96%) and good specificity (70%). These findings are important as they suggest it may be possible to retain excellent project-level sensitivity with fewer crowd assessments per citations. Reducing the number of assessments per citation could have multiple advantages, including reducing the time to individual project completion, increasing the number of projects a crowd of set size can assist with, and maximizing work performed (specificity). Although most crowd members performed well, 4% of the crowd were observed to have less than adequate sensitivities (<80%). Although it only represents a minority, inclusion of 1 or more of these poorly performing crowd members could place a project at risk if the number of assessments per citation was significantly reduced. Although the goal in our study was to evaluate the performance of an uncurated crowd, we acknowledge that it would have been reasonable and potentially beneficial to attempt the removal of these poorly performing individuals by requiring the successful completion of a test set. This approach was employed in the 2 crowdsourcing studies utilizing the Amazon Mechanical Turk system, where workers were required to successfully evaluate 3 articles before being invited to the full project [14,22]. Although this approach would have had benefits, the authors did also observe that initial testing alone was insufficient as some reviewers developed “unconscientious” behavior over time that required embedded quality control or “honey pots” [22]. Available evidence suggests that with the right combination of initial testing and ongoing monitoring, it will be possible to further optimize crowd sensitivity and work performed [39,40]. It will be important for future studies to establish the initial and embedded testing required to guarantee comprehensive SRs, while optimizing crowd work. The size and components of the test set, the threshold for sensitivity, and how to embed quality control will need to be evaluated as part of larger studies.

### Crowdsourcing—Barriers to Implementation and Future Directions

Although crowdsourcing has the potential to lead to more rapid knowledge synthesis and evidence translation, it is important to acknowledge that it can only do so if accessible, cost-effective, and scalable. Presently, and similar to what transpires in other areas of interventional and diagnostic research, the innovation is initially only available to a handful of individual teams and organizations who have taken the considerable time to both develop a platform (Cochrane Crowd, CrowdscreenSR) or adopt one (Amazon mTurk) and recruit a crowd. Although it may not be possible for other SR teams requiring a crowd for a large project to access the exact individual or crowds utilized in the existing feasibility and validation studies, overall findings do suggest it would be possible to rapidly recruit a similarly sized and motivated crowd through emails and promotional materials. Consider, for example, that each major center in North America, and beyond, has hundreds potentially thousands of undergraduate medical students, residents, and health care professionals who may want to engage in knowledge synthesis efforts. Although some large institutions and organization, similar to Cochrane, may consider creating their own SR crowdsourcing initiative, there are considerable costs associated with the development and maintenance of a user-friendly robust platform that allows investigators to present projects and both evaluate and track crowd performance through the citation review process. Consequently, the ideal future state includes the development of an online SR citation screening platform broadly available to a wide range of institutions, organization, and countries that share both the costs and benefits of the platform. As the success of such an initiative would necessitate engaging with a large online distributed crowd with a broad range of interest and experiences, future work in this area should seek to understand what motivates individuals to assist with crowdsourcing SR tasks [41]. It is worth noting that the aforementioned SR crowdsourcing studies and initiatives have been able to succeed using motivators such as certain types of payment and volunteer or research experience. Whether these would be sufficient on a large scale remains to be determined. Missing from this list are more objective measures of academic credit, including group or named authorship. Although not part of our original study protocol, many of the individual crowd members expressed interest in, and have since participated in, SRs for named authorship or as part of a group at our institute [42].

### Crowdsourcing and Machine Learning

Future work aimed at developing a platform capable of facilitating and optimizing crowdsourcing into SR should also consider incorporating automated or computerized abstract screening. This has been hypothesized and investigated as an alternative means of reducing the work required by SR investigative teams. The findings in our crowdsourcing study and those reported by Mortensen [35] are similar to or exceed the 30% to 70% reported in text-mining studies. Machine learning has shown strong accuracy and cost-effectiveness when studies have focused on a single screening criterion (ie, study design—RCT or not). Where multiple elements of the articles need to be assessed, machine learning can require considerable costs related to training. Although a comparison of crowdsourcing with text-mining performance is valid, it is also worthwhile considering that by combining machine learning and crowdsourcing together may lead to the greatest workload reduction for the crowd and investigative teams [43,44]. This hybrid approach has been researched and applied in a variety of fields outside the SR field. As an immediately relatable example, Google employs machine learning to generate search results, which are then further improved by integrating users’ selection [45]. For SR screening, this combined approach would involve having the machine learn on an initial training set prepared by the investigative team, followed by identification of very low probability citations, using machine learning, with the remaining referred to the crowd. The lone published study to consider this approach by Wallace et al had a machine-learning algorithm to identify citations unlikely to be an RCT. This approach eliminated 80% of the citations, with the remaining 20% containing 98% of the eligible citations [43]. Interestingly, the authors estimated that this approach could reduce study costs by 7-fold. Another approach proposed by Bannach-Brown would be to use crowd’s assessments on a training set to develop the machine-learning approach, which would be later reused on the training set (to identify potential errors) or the remaining of citations [46].

### Study Strengths and Limitations

This study offers a significant contribution to the emerging field of SR crowdsourcing. It is the first to report on crowd members’ demographics, their capacity for full text retrieval, and performance on evaluating full text. However, certain limitations must be acknowledged. First, although our study provides evidence supporting the feasibility of crowdsourcing, the platform used for the study and the exact crowd are no longer available. Fortunately, recruitment of a sufficiently sized crowd (>30) allows us to provide a reliable estimate of the performance of the much larger population of individuals with science or health training distributed across centers and cities around the world who might consider participating in SR projects. Second, demographic information collected on the crowd participants determined that the majority were from the Ottawa region and had some postsecondary education. As demographic and training data were self-reported, we cannot be certain of the accuracy of these data and the implications of misclassification (eg, falsely elevating experience). Given the uncertainty about both the accuracy of self-reported education and training data and generalizability of the results to more geographically diverse crowds, we would recommend that scientists and clinicians incorporate initial and embedded quality control measures in an SR crowdsourcing initiative. Third, although our study suggests that it may be possible, perhaps beneficial, to consider fewer crowd assessments per citation, in the setting of initial and ongoing testing, it is not yet possible to provide definitive guidance. Fourth, although this study evaluated crowd performance on citations from 6 different SRs, the largest to date, it is not yet clear how well the results extrapolate to reviews from divergent areas. Our findings would generalize best to SR focused on health and those focused on children. Finally, this study was not properly designed to evaluate or comment on cost or time saving. As an early exploratory pilot work that required the development of a software platform and crowd recruitment, it is likely that no benefit would have been observed. We would suggest that this work be reserved for after the development of the aforementioned user-friendly robust online platform and recruitment of a sufficiently sized motivated crowd.

### Conclusions

This study supports the feasibility and validity of crowdsourcing as a means to facilitate citation screening for SRs by a crowd of nonexpert volunteers with some medical and/or scientific background. It also offers the first evidence for screening at the full text level. This approach is not intended to replace the gold standard expert screening but rather to supplement it by expediting the screening process, thus allowing the investigative team to focus on more complex SR tasks. To get the full potential benefits of crowdsourcing, future projects should aim at establishing a comparable platform that would allow researchers to easily access a large and expanding crowd similar to the one recruited here. Future directions should assess the motivation of the crowd, what incentives could improve performance, how to predict the crowd members with higher performance, and the need for quality control measures such as honeypots.

## Appendix

Multimedia Appendix 1 Privacy policy and terms of use.

Multimedia Appendix 2 Standards for Reporting of Diagnostic Accuracy Studies guidelines.

Multimedia Appendix 3 Promotional material.

Multimedia Appendix 4 Citations disposition based on crowd’s assessment using multiple exclusion thresholds.

Multimedia Appendix 5 Number of assessments per paper at each screening level.

Multimedia Appendix 6 Crowd’s sensitivity and work performed by systematic review at different exclusion thresholds for the abstract level.

Multimedia Appendix 7 Crowd’s sensitivity and work performed by systematic review at different exclusion thresholds.

Multimedia Appendix 8 Individual crowd members’ performance including those who completed less than 50 assessments.

Multimedia Appendix 9 Individual crowd members’ performance by systematic review.

## Abbreviations

3D: three-dimensional
CHEO: Children’s Hospital of Eastern Ontario
IQR: interquartile range
RCT: randomized controlled trial
SR: systematic review
