# Supplementary material for: Crowdsourcing the Citation Screening Process for Systematic Reviews: Validation Study
Source: J Med Internet Res. 2019 Apr 29;21(4):e12953. doi: 10.2196/12953 (PMC6658317; doi:10.2196/12953)
Supplement: Multimedia Appendix 1 [file jmir_v21i4e12953_app1.pdf]

Terms of Use

Last updated: April 27, 2016

Introduction

Welcome to CrowdScreen. CrowdScreen is a software platform designed to assist with knowledge synthesis and systematic review. The CrowdScreen platform is provided through a web application. Dayre McNally and Nassr Nama developed CrowdScreen and have given the Children's Hospital of Eastern Ontario Research Institute (CHEO RI) permission and license to use and administer the platform. The "we", "us" or "our" used in this document refers to Dayre McNally, Nassr Nama, and the CHEO RI.

This document describes the terms and conditions that govern your use of this website. By using this website, you accept these terms and conditions in full. If you disagree with any part of these terms and conditions you must not use this website.

Privacy policy

A privacy policy has been developed for the website. This policy will tell you what information we may collect about you, what we use it for, and who we share it with.

User accounts

To access the platform you will need to request and create an account. Your login may only be used by you. You are responsible for the activity on your account, including the content that is uploaded. It is up to you to keep your password secure, and to notify us immediately if any unauthorised access of your account occurs. We are not liable for any losses or other consequences of unauthorised use of your account.

Use of the platform

Project leads and principal investigators may request permission to use the website to facilitate systematic reviews or related knowledge synthesis projects. Others users, may use their account to request permission to assist with a project hosted on the website. If provided, permission gives license to access and use the platform for its intended purpose. This license is personal to you and may not be assigned or sub-licensed to anyone else.

All platform users agree not to:

- Use this website in any way that causes, or may cause, damage to the website or impairment of the availability or accessibility of the website;
- Use the website in any way which is unlawful, illegal, fraudulent or harmful, or in connection with any unlawful, illegal, fraudulent or harmful purpose or activity.
- Use this website to copy, store, host, transmit, send, use, publish or distribute any material which consists of (or is linked to) any spyware, computer virus, Trojan horse, worm, keystroke logger, rootkit or other malicious computer software.
- Conduct any systematic or automated data collection activities (including without limitation scraping, data mining, data extraction and data harvesting) on or in relation to this website without our express written consent.
- Use this website to transmit or send unsolicited commercial communications.
- Use this website for any purposes related to marketing without the express written consent of Dayre McNally and Nassr Nama.
- Reverse engineer the platform or access the platform to copy any features or functions of the platform.
- Attempt to access a project or systematic review by any means other than invitation by the review principal investigator (or their delegate).

Platform availability

Access to CrowdScreen is granted at our discretion and permission to use the site can be withdrawn at any time.

We will try to maintain a secure application, but we do not promise it will be error or problem free. Third party vendors and hosting partners are used to provide the hardware, software, networking, storage and related technology required to run the platform. The platform is provided on an “as is” and “as available” basis. We do not guarantee that use of it will be uninterrupted, timely, secure or error-free. At any time we may modify or discontinue (temporarily or permanently) the platform with or without notice. We are not liable to you or any third party for any such modification, suspension or discontinuance. **To protect against the loss of data, project leads and investigator teams should frequently export the data generated related to their project.**

Restricted access

Access to certain areas of this website is restricted. We reserve the right to restrict access to specific areas of this website, or indeed this entire website, at our discretion. If we provide you with a user ID and password to enable you to access a restricted area of this website or other content or services, you must ensure that the user ID and password are kept confidential.

User content

In these terms and conditions, “your user content” means material (including without limitation text, images, audio material, video material and audio-visual material) that you submit to this website, for whatever purpose.

We support and respect the intellectual property rights of others. You are solely responsible for your own content – for its accuracy, quality, integrity and legality – and the consequences of submitting and publishing it on the platform. You must own or have the rights, licenses, consents and permissions to upload and publish content on the platform. In submitting content, or publishing on the site, you confirm that you do have these rights and that your content does not infringe any third party copyright or other proprietary rights. We expect you to have the rights to publish your content, and for it to be appropriate.

You must not upload, store or transmit content that is in itself is, or encourages behaviour that is:

- Sexually explicit or pornographic
- Violent or cruel to humans or to animals
- Hateful or discriminatory
- Exploiting of minors
- Fraudulent or illegal, including defamation
- In violation of third party privacy rights

CrowdScreen is not responsible for the topic or content of a review, including its accuracy, appropriateness or whether it is in breach of intellectual property rights. Please inform us if you find questionable content. We reserve the right to edit or remove any material submitted to this website, or stored on servers, or hosted or published upon this website. Notwithstanding our rights under these terms and conditions in relation to user content, we do not undertake to monitor the submission of such content to, or the publication of such content on, this website.

By creating a user account and contributing content you grant us worldwide, irrevocable, non-exclusive, royalty-free license to use, reproduce, adapt, publish, translate and distribute your user

content. We require this license to display user content, yours and those working together on a project, to you on CrowdScreen website. This license also allows us to evaluate user content with the goal of improving the service provided by the platform and to complete and publish research studies related to the service provided by the platform. You understand that we do not guarantee confidentiality with respect to the content that you submit.

Relationship between users

CrowdScreen is a platform where users can work together on knowledge synthesis and systematic reviews. Users who sign up to assist with a project do so voluntarily. By logging in and submitting content they are giving the project lead and CrowdScreen permission to not only view and use, but ownership over the content.

CrowdScreen recognizes that there may be other reasons why users may consider assisting with project, including education (course work), recognition in the form of volunteer time or authorship or payment (i.e. case, gift cards, other). The CrowdScreen platform, its owners or operators, intentionally does not provide a service to mediate these agreements. We will not entertain request to establish or manage agreements between users, and will not help resolve any conflicts that develop.

Suspension of accounts and projects

Users identified as project leads may request that a project be removed from the platform. Further we may remove a project from the platform at our sole discretion without notice or explanation.

All users are able to inactivate their account, and by doing so this remove their ability to login and complete micro-tasks. Similarly we may inactivate an account at our sole discretion without notice or explanation.

Content submitted by the user prior to inactivation is retained as the property of the project lead and CrowdScreen.

Warranty and liability

This website is provided “as is” without any representations or warranties, express or implied. We make no representations or warranties in relation to this website or the information and materials provided on this website. You understand that we are not liable for any direct, indirect, incidental, special, consequential or exemplary damages, including but not limited to, damages for loss of profits, goodwill, use, data or other intangible losses resulting from:

(i) the use or the inability to use the platform;

(ii) the content posted on the platform;

(iii) unauthorized access to or alteration of your content;

(iv) statements or conduct of any third party on the platform;

(v) or any other matter relating to the platform.

Nothing on the website constitutes, or is meant to constitute, advice of any kind.

Indemnity

You hereby indemnify, defend and hold harmless Dayre McNally, Nassr Nama, CHEO and the CHEO RI from and against any and all losses, damages, costs, debts, claims, suits, proceedings and expenses arising from:

(i) your use of and access to the platform;

(ii) your violation of any term of these terms of use

(iii) your violation of any third party right, including without limitation any copyright, property, or privacy right;

(iv) any claim that your content caused damage to a third party.

This defence and indemnification obligation will survive these terms of use and your use of the platform.

Unenforceable provisions

If any provision of this website disclaimer is, or is found to be, unenforceable under applicable law, that will not affect the enforceability of the other provisions of this website disclaimer.

Change to terms

We may revise these terms and conditions from time-to-time, at our discretion. You accept the new terms with continued use of the platform. Revised terms and conditions will apply to the use of this website from the date of the publication of the revised terms and conditions on this website. Please check this page regularly to ensure you are familiar with the current version. If in our opinion the modification represents a material change to the terms, we will notify you by posting an announcement.

Intellectual Property and Assignment

We may transfer, sub-contract or otherwise deal with their rights and/or obligations under these terms and conditions without notifying you or obtaining your consent. You may not transfer, sub-contract or otherwise deal with your rights and/or obligations under these terms and conditions.

Governing Law

These terms and conditions are governed by and construed in accordance with the laws of Canada

Breaches of these terms and conditions

If you breach these terms and conditions in any way, we may take such action as we deem appropriate to deal with the breach, including suspending your access to the website, prohibiting you from accessing the website, blocking computers using your IP address from accessing the website, contacting your internet service provider to request that they block your access to the website and/or bringing court proceedings against you.

Privacy Policy

Last updated: April 27, 2016

This privacy policy has been compiled to better serve those who are concerned with how their Personally identifiable information is being used by the CrowdScreen website. Personal identifiable information is information that can be used on its own or with other information to identify, contact, or locate a single person, or to identify an individual in context.

We respect your privacy and are committed to protect the personal information of those who use the CrowdScreen site – investigators, reviewers and visitors browsing the site ("Users"). We believe that you have the right to know our practices regarding the information we may collection when you visit and/or use our website.

Please read this document to understand our policies and practices regarding your information. This page informs you of our policies regarding the collection, use and disclosure of Personal Information we receive from users of the Site. By using the Site, you agree to the collection and use of information in accordance with this policy. If you do not agree with our policies and practices, your choice is not to use the site.

Children

This website is not intended for children under 13 years of age. No one under age 13 may provide any personal information to or on the site. We do not knowingly collect personal information from children under 13. If you are under 13, do not use or provide any information on this website. If we learn that personal information has been collected from a children under 13 we will delete that information.

Information Collected

While using our Site, we may ask you to provide us with certain personal information. (1) When you register to the site you are asked to provide certain personal information including, but not limited to, a valid email address, full name, username and password. (2) On the Profile page we also allow you to provide additional personal information such as, but not limited to, an online name, your age, education/training, and previous research experience.

In addition, we collect and generate information about you while you access, browse, view and otherwise use the site. This may include, but is not limited to, filling out forms, completing a survey or entering information on our site. When you access the site we are aware of your usage of the site and may gather, collect and record information related to such usage. For example, we may use the information provided during the assessments of citations (asbtracts or full text) to determine how well you perform at the tasks you have chosen to undertake.

Once you register, your username, and additional information you provide on the profile page, and information generated based on your website activity may be made visible to the other users of the site.

How do we collect information

Most of the information collected is provided directly by the user. You will do this by filling out the registration details and by posting and sharing additional information voluntarily. Like many site operators, we may also collect information that your browser sends whenever you visit our Site("Log Data"). This Log Data may include information such as your computer's Internet Protocol ("IP") address, operating system, browser type, browser version, the pages of our Site that you visit, the time and date of your visit, the time spent on those pages and other statistics. In addition, we may use third party services that collect, monitor and analyze this information.

How do we use the information?

We collect this information to provide a quality service, enhance the experience of all users, as well as to monitor the site for fraud and inappropriate content or behavior.

More specifically, we may use the information collected from you to verify your identity. We may also use this information to establish and set up your account, re-issue a password, log your activity and contact you. This information may help us improve our service to you. For example, with the information collected we may be able to identify new projects that may be of interest to you. As another example, the information generated from your assessment of citations may help us identify fraudulent activities and another inappropriate activity. We will ask for your consent before using information for a purpose other than those set out in this privacy policy.

Should you ever deactivate your account we will keep your information on file as long as required comply with regulatory requirements and as long as it is necessary and relevant for our operations and the completion of projects to which you contributed. In addition, we may retain personal information from closed accounts to comply with legal requirements prevent fraud, collect any fees owed, resolve disputes, troubleshoot problems, assist with any investigation, enforce our Site terms and take other actions permitted by law.

Sharing of Personal Information with Third Parties

We will not sell, trade, or otherwise transfer to outside parties your personally identifiable information. This does not include website hosting partners and other parties who assist us in operating our website or serving our users, so long as those parties agree to keep this information confidential. We may also release information when its release is appropriate to comply with the law, enforce our site policies, or protect ours or others' rights, property, or safety.

Communications

We may use your Personal Information to contact you with newsletters or to contact you about new systematic reviews or evidence based medicine initiates that may interest you.

Security

The security of your Personal Information is important to us. It is important to remember that no method of transmission over the Internet, or method of electronic storage, is 100% secure. Your personal information is contained behind secured networks and is only accessible by a limited number of persons who have special access rights to such systems, and are required to keep the information confidential. While we will strive to protect your Personal Information, we cannot guarantee its absolute security. Users who have registered to the site agree to keep their password in strict confidence and not disclose it to a third party.

Cookies

We do not use cookies for tracking purposes

Third Party Sites

While using the site you may encounter links to a third party website. CrowdScreen is not responsible for these sites and has no responsibility or liability whatsoever with regard to privacy matters or any other legal matter with respect to such sites. We encourage you to carefully read the privacy policies and terms of use or service of those sites.

Changes to This Privacy Policy

This Privacy Policy is effective as of April 27, 2016 and will remain in effect except with respect to any changes in its provisions in the future, which will be in effect immediately after being posted on this page. We reserve the right to update or change our Privacy Policy at any time and you should check this Privacy Policy frequently. Your continued use of the Service after we post any modifications to the Privacy Policy on this page will constitute your acknowledgment of the modifications and your consent to abide and be bound by the modified Privacy Policy. If we make any material changes to this Privacy Policy, we will notify you either through the email address you have provided us, or by placing a prominent notice on our website.

Contact Us

If you have any questions about this Privacy Policy, please contact us.

James Dayre McNally

Children's Hospital of Eastern Ontario

401 Smyth Road, Ottawa Ontario

dmcnally@cheo.on.ca
