# Supplementary material for: Crowdsourcing the Citation Screening Process for Systematic Reviews: Validation Study
Source: J Med Internet Res. 2019 Apr 29;21(4):e12953. doi: 10.2196/12953 (PMC6658317; doi:10.2196/12953)
Supplement: Multimedia Appendix 3 [file jmir_v21i4e12953_app3.pdf]

# WANTED

## DO YOU HAVE 60 SECONDS?

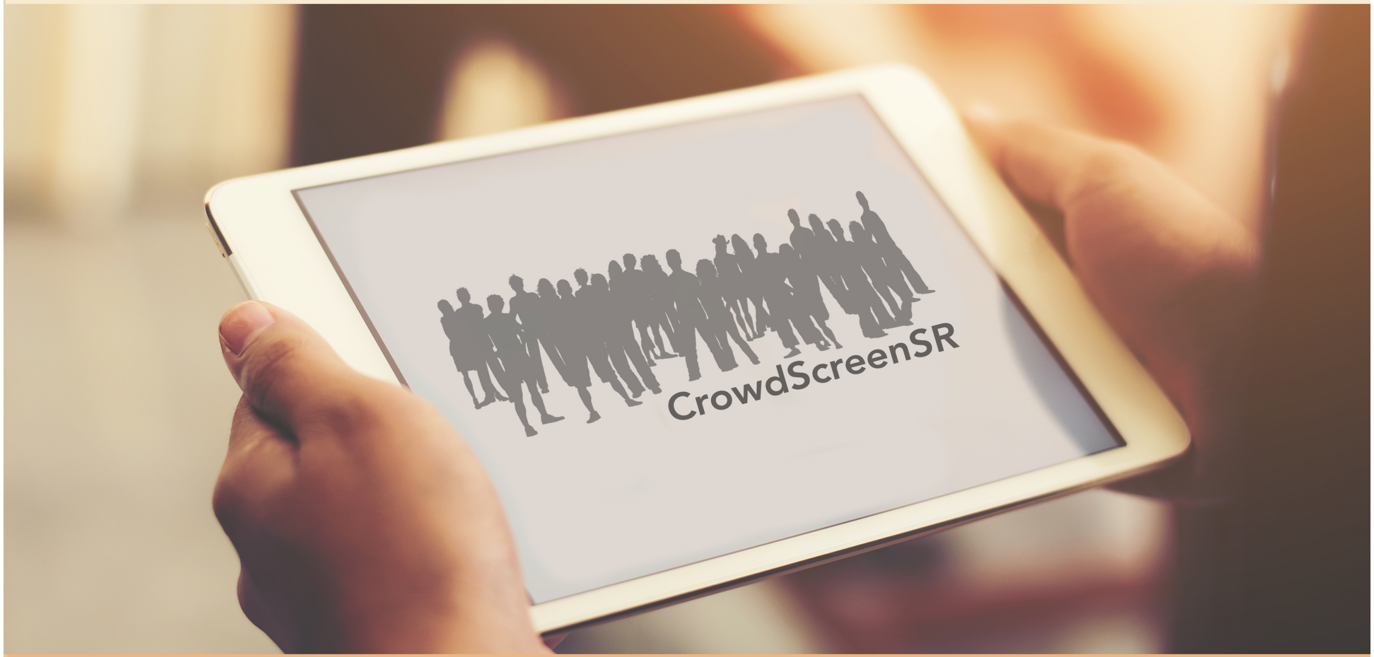


## DO YOU ENJOY READING LITERATURE AND ANSWERING RESEARCH QUESTIONS?

**THE PROBLEM**: Systematic reviews are an essential part of medicine. By summarizing all available knowledge on a topic the can change the way patients receive care and guide new research. Unfortunately, due to the rapid rise in scientific literature it is increasingly challenging to complete these reviews.

**THE SOLUTION:** Build a bigger team (crowdsource the task).

**OUR ASK:** Sign-up to help test this solution. Less than one minute is all it takes to evaluate an article.

## WHAT'S IN IT FOR YOU?

Reimbursement (e.g. Authorship, gift cards) Join a research team
Obtain research experience Letters of reference
Help answer a research question

**www.cheori.org/en/crowdscreenproject**

# CrowdScreenSR Research Study
# http://www.cheori.org/en/crowdscreenproject

## What problem does this study hope to address?

Systematic reviews are an essential part of medicine as they seek to summarize everything known about a topic. The answers provided are used to change the way patients receive care and identify areas where further research is needed. Systematic reviews are usually completed by a small group of experts, who must assess hundreds or thousands of studies to find the few that are relevant (about 3 in 100 are relevant). It can take many months, sometimes years, to complete this task. Unfortunately, due to the rapid rise in the number of published studies, it is more challenging than ever for the small group of experts to perform a comprehensive review in a timely fashion.

Our CHEO research team is interested in exploring whether it is possible for members of the broader university and medical community to help topic experts complete their systematic review.

## Can you help?

Yes. We need help from a wide variety of community members. If you have medical training or previous research experience - great. If you do not have medical or prior research experience - also great.

## How to participate?

**Step 1** (sign up) - Please begin by signing up. Log-into the CrowdScreenSR website here.

**Step 2** (demo) - The demo will help familiarize you with the website and show you how to find available reviews and how to assess whether a citation is relevant.

**Step 3** (available reviews) - Once logged in to the website you can read and learn about REAL systematic reviews recently completed at CHEO. Pick one that interests you and complete the Test Set.

**Step 4** (Test Set) - You will be presented with the title and abstract of 10 potentially eligible studies. An abstract is a concise summary of the article provided by the authors (approximately 300 words in length). After reading the title and abstract, you will then decide whether it meets the eligibility criteria provided by the investigators. During the demo you will receive feedback on whether your assessment was correct.

**Step 5** (start reviewing the studies) - Once you have completed the demo you can request access to the full set of articles. You will be asked to evaluate a minimum of 100 and up to 500 articles. Screening the minimum of 100 abstracts is a fairly quick task that can be completed in 1-2 hours!

## Why participate?

There are many reasons you could choose to participate. First, you will help answer an important research question. Second, you will gain knowledge in a field of your interest by reading about articles related to that subject. Third, you will learn about systematic reviews and you can list this as a research experience on your CV. Fourth, for those who complete all study procedures (related to their systematic review) we are willing to provide a letter detailing your involvement. Fifth, participants can choose to be notified about future systematic reviews that use the CrowdScreenSR website and are looking for help (some of these groups may provide compensation).

## Will I be compensated?

In the future it is possible that some investigators may choose to compensate individuals for assisting with a systematic review (e.g. authorship, $$). As the systematic reviews we are using for this study are completed, it is not possible for study participants to be an author or be paid by the principal investigator. We recognize that study participants are performing a valuable task, and to provide some incentive for highly quality work, we will be offering gift cards (up to $100) for the participants that have the most number of correct answers (three per systematic review).

If you have any questions, feel free to email our team at crowdscreenteam@gmail.com.
