# Supplementary material for: Crowdsourcing the Citation Screening Process for Systematic Reviews: Validation Study
Source: J Med Internet Res. 2019 Apr 29;21(4):e12953. doi: 10.2196/12953 (PMC6658317; doi:10.2196/12953)
Supplement: Multimedia Appendix 4 [file jmir_v21i4e12953_app4.pdf]

Multimedia Appendix 4. Citations disposition based on crowd's assessment using multiple exclusion thresholds.

Retain	Exclude	> 50%	> 75%	= 100%
4	0	Retain	Retain	Retain
3	1	Retain	Retain	Retain
2	2	Retain	Retain	Retain
1	3	Exclude	Retain	Retain
0	4	Exclude	Exclude	Exclude
