# Supplementary material for: Crowdsourcing the Citation Screening Process for Systematic Reviews: Validation Study
Source: J Med Internet Res. 2019 Apr 29;21(4):e12953. doi: 10.2196/12953 (PMC6658317; doi:10.2196/12953)
Supplement: Multimedia Appendix 5 [file jmir_v21i4e12953_app5.pdf]

Multimedia Appendix 5. Number of assessments per paper at each screening level.

	Abstract Level			Full Text Level		
SR	N [a]	Median (IQR)	Range	N [a]	Median (IQR)	Range
Anesthesiology	243	8 (8 – 9)	8 – 10	203	7 (7 – 7)	5 – 9
Cardiology	486	8 (8 – 9)	7 – 10	141	17 (17 – 18)	16 – 20
Emergency	482	8 (7 – 8)	7 – 9	83	11 (10 – 11)	9 – 12
Endocrinology	200	9 (7 – 9)	7 – 10	67	15 (15 – 15)	8 – 17
Respirology	256	8 (8 – 8)	8 – 9	150	6 (6 – 6)	5 – 7
Surgery	527	6 (6 – 6)	5 – 7	283	3 (2 – 3)	2 – 4
Overall	2194	8 (7 – 8)	5 – 10	927	7 (3 – 11)	2 – 20

[a] N represents the number of papers assessed at this level.
