# Supplementary material for: Crowdsourcing the Citation Screening Process for Systematic Reviews: Validation Study
Source: J Med Internet Res. 2019 Apr 29;21(4):e12953. doi: 10.2196/12953 (PMC6658317; doi:10.2196/12953)
Supplement: Multimedia Appendix 6 [file jmir_v21i4e12953_app6.pdf]

Multimedia Appendix 6. Crowd's sensitivity and work-saved by systematic review at different exclusion thresholds for the abstract level [a-c].

Crowd agreement required to exclude	= 100%		> 75 %		> 50%	
	Sensitivity	Work-Saved	Sensitivity	Work-Saved	Sensitivity	Work-Saved
Anesthesiology	100.0	22.3	100.0	32.3	100.0	42.0
Cardiology	100.0	49.8	100.0	71.2	98.6	76.7
Emergency	100.0	65.0	100.0	83.5	100.0	90.5
Endocrinology	100.0	55.2	100.0	66.7	100.0	74.6
Respirology	100.0	29.8	100.0	43.4	95.7	52.8
Surgery	100.0	37.9	100.0	49.8	100.0	59.0
Overall	100.0	44.9	100.0	60.1	98.9	68.0

[a] Citations were excluded based on different thresholds. Sensitivity and work-saved were measured after abstract screening. A citation was excluded if the percentage of assessments that excluded the paper at the abstract level was higher than the specified threshold.

[b] Sensitivity is the percentage of eligible citations, identified by the experts, that were retained by the crowd.

[c] Work-saved is the percentage of citations that were excluded by the crowd and did not require assessment by the investigative team at abstract level.
