# Supplementary material for: Crowdsourcing the Citation Screening Process for Systematic Reviews: Validation Study
Source: J Med Internet Res. 2019 Apr 29;21(4):e12953. doi: 10.2196/12953 (PMC6658317; doi:10.2196/12953)
Supplement: Multimedia Appendix 8 [file jmir_v21i4e12953_app8.pdf]

Multimedia Appendix 8. Individual crowd members’ performance including those who completed less than 50 assessments [a].

	Abstract Level (N=53)		Full Text Level (N=74)		Overall (N=118) [b]	
	Median (IQR)	Range	Median (IQR)	Range	Median (IQR)	Range
Assessments	234 (43 – 485)	1 – 2194	54 (12 – 141)	1 – 786	90 (20 – 251)	1 – 2980
Sensitivity [c] (%)	97.2 (92.3 – 100.0)	55.0 – 100.0	97.3 (89.6 – 100.0)	0.0 – 100.0	97.2 (90.2 – 100.0)	0.0 – 100.0
Specificity [d] (%)	76.0 (67.4 – 93.1)	40.0 – 100.0	64.3 (50.6 – 80.3)	0.0 – 100.0	69.4 (58.0 – 89.5)	0.0 – 100.0

[a] Results are provided per crowd member.

[b] Total number of reviewers does not equal the sum of both levels as some reviewers participated in both.

[c] Sensitivity is the percentage of eligible citations, identified by the experts, that were retained by the crowd member. It is based on 43 crowd members at the abstract level, 63 at the full text level and 114 overall. The remaining crowd members did not assess any eligible citations.

[d] Specificity is the percentage of ineligible citations, as discarded by the experts, that were also excluded by the crowd member.
