# Supplementary material for: Crowdsourcing the Citation Screening Process for Systematic Reviews: Validation Study
Source: J Med Internet Res. 2019 Apr 29;21(4):e12953. doi: 10.2196/12953 (PMC6658317; doi:10.2196/12953)
Supplement: Multimedia Appendix 9 [file jmir_v21i4e12953_app9.pdf]

Multimedia Appendix 9. Individual crowd members' performance by systematic review [a,b].

SR	Reviewers	Total Assessments	Assessments per reviewer	Sensitivity [c]	Specificity [d]
	N	N	Median (IQR)	Median (IQR)	Median (IQR)
Anesthesiology	19	3507	126 (70 – 256)	98.3 (94.9 – 100.0)	60.9 (56.4 – 68.1)
Cardiology	32	6547	141 (135 – 250.5)	97.2 (86.5 – 99.3)	65.9 (56.9 – 87.0)
Emergency	21	4460	107 (83 – 251)	100.0 (85.7 – 100.0)	94.6 (83.8 – 96.3)
Endocrinology	21	2418	67 (66 – 152)	96.7 (90.0 – 96.7)	82.5 (71.6 – 88.9)
Respirology	13	2875	256 (149 – 256)	93.8 (87.0 – 100.0)	60.0 (58.5 – 63.0)
Surgery	11	3677	287 (105 – 527)	100.0 (93.8 – 100.0)	58.5 (56.8 – 82.0)
Overall	117	23484	141 (83 – 256)	96.7 (89.7 – 100.0)	71.4 (59.6 – 88.9)

[a] Only crowd members who have completed 50 assessments or more in one review were included in this table.
[b] Results are provided per crowd member.
[c] Sensitivity is the percentage of eligible citations, identified by the experts.
[d] Specificity is the percentage of ineligible citations, as discarded by the experts, that were also excluded by the crowd member.
